# Delays in starting antiretroviral therapy in patients with HIV-associated tuberculosis accessing non-integrated clinical services in a South African township

**DOI:** 10.1186/1471-2334-11-258

**Published:** 2011-09-30

**Authors:** Stephen D Lawn, Lucy Campbell, Richard Kaplan, Francesca Little, Carl Morrow, Robin Wood

**Affiliations:** 1 The Desmond Tutu HIV Centre, Institute for Infectious Disease and Molecular Medicine, Faculty of Health Sciences, University of Cape Town, Cape Town, South Africa; 2 Department of Clinical Research, Faculty of Infectious and Tropical Diseases, London School of Hygiene & Tropical Medicine, London, UK; 3 Department of Statistical Sciences, Faculty of Science, University of Cape Town, South Africa; 4 School of Public Health and Family Medicine, Faculty of Health Sciences, University of Cape Town, Cape Town, South Africa

## Abstract

**Background:**

Delays in the initiation of antiretroviral therapy (ART) in patients with HIV-associated tuberculosis (TB) are associated with increased mortality risk. We examined the timing of ART among patients receiving care provided by non-integrated TB and ART services in Cape Town, South Africa.

**Methods:**

In an observational cohort study, we determined the overall time delay between starting treatment for TB and starting ART in patients treated in Gugulethu township between 2002 and 2008. For patients referred from TB clinics to the separate ART clinic, we quantified and identified risk factors associated with the two component delays between starting TB treatment, enrolment in the ART clinic and subsequent initiation of ART.

**Results:**

Among 893 TB patients studied (median CD4 count, 81 cells/μL), the delay between starting TB treatment and starting ART was prolonged (median, 95 days; IQR = 49-155). Delays were shorter in more recent calendar periods and among those with lower CD4 cell counts. However, the median delay was almost three-fold longer for patients referred from separate TB clinics compared to patients whose TB was diagnosed in the ART clinic (116 days versus 41 days, respectively; P < 0.001). In the most recent calendar period, the proportions of patients with CD4 cell counts < 50 cells/μL who started ART within 4 weeks of TB diagnosis were 11.1% for patients referred from TB clinics compared to 54.6% of patients with TB diagnosed in the ART service (P < 0.001).

**Conclusions:**

Delays in starting ART were prolonged, especially for patients referred from separate TB clinics. Non-integration of TB and ART services is likely to be a substantial obstacle to timely initiation of ART.

## Background

HIV-associated tuberculosis (TB) is associated with high mortality risk and accounts for approximately 25% of global HIV/AIDS deaths [1]. In addition to appropriate antituberculosis treatment, case management requires administration of trimethoprim-sulphamethoxazole prophylaxis, which halves mortality risk [2], and antiretroviral therapy (ART), which reduces mortality by 64%-95% [3]. Although ART greatly improves survival, the optimal time to start ART during TB treatment has, for a long time, remained unclear. However, cumulative findings from observational studies and more recent randomized controlled trials have demonstrated that overall delays in ART initiation are associated with increased mortality among TB patients across a wide spectrum of baseline CD4 cell counts [4-7].

In response to these data, World Health Organization (WHO) ART guidelines have been updated on several occasions between 2002 and 2010, recommending progressively more rapid initiation of ART during TB treatment [4]. The most recent revision of these guidelines published in 2010 recommend that ART should be given to all patients regardless of CD4 cell count and should be started as soon as possible after TB treatment is tolerated and not later than 8 weeks after commencement of TB treatment [8]. Data from subsequent randomised controlled trials show that patients with CD4 cell counts < 50 cells/μL have a particularly high mortality risk and should receive ART within 2 weeks [9-11].

Despite these policy changes, the operational feasibility of rapid initiation of ART in patients accessing routine services in resource-limited settings is not known. In particular the operational challenge to achieving this within the context of separate non-integrated TB and ART clinical services has not been assessed. In this study we therefore quantified and explored determinants for the delay between starting TB treatment and starting ART among TB patients enrolling in a large township in Cape Town, South Africa, between 2002 and 2008. We discuss the major obstacle that non-integration of TB and ART services is likely to represent with regard to implementation of the 2010 WHO guidelines for the timing of ART.

## Methods

### Antiretroviral treatment cohorts

The Hannan Crusaid ART clinic in Gugulethu township, Cape Town, has provided ART free of charge since 2002. Consistent with the South African national guidelines [12], patients with WHO stage 4 disease (AIDS) and all those with blood CD4 cell counts < 200 cells/μL were eligible for treatment. The huge burden and complications of TB in this ART service have been previously described [13-16]. Here, as in most of South Africa, TB treatment and ART have been delivered by separate primary care clinics in different localities within the township. Thus, patients with HIV-associated TB were referred to the ART clinic from separate TB clinics [15]. Other patients were referred to the ART clinic from antenatal clinics, sexual health clinics, general medical out-patient clinics or following discharge from an in-patient admission.

TB cases were diagnosed and notified according to South African TB programme guidelines and TB was treated using standardised rifampicin-based regimens of 6 months duration for new TB cases and 8 months for retreatment cases. WHO guidelines during the period of the study recommended that TB patients with CD4 cell counts < 200 cells/μL should start ART within the first 2-8 weeks of TB treatment [17,18]. However, South African ART guidelines first issued in 2004 recommended that patients with CD4 cell counts < 50 cells/μL or with serious co-morbidity start ART after 2 weeks of TB treatment and that those with CD4 cell counts of 50-200 cells/μL start ART after 8 weeks.

### Data sources

With ethical approval from the Research Ethics Committee of the University of Cape Town and written informed consent from all patients, a prospective clinical database has been maintained on patients enrolling in this ART service. All patients enrolling between September 2002 and January 2008 were considered for inclusion in this analysis. Eligible patients were ART-naive, aged over 18 years, had a current notified diagnosis of TB and were receiving antituberculosis therapy during the pre-ART screening period. For each patient, demographic details, TB classification (new, retreatment, pulmonary and extrapulmonary), WHO clinical stage, blood CD4 cell counts and dates of TB diagnosis and ART initiation were recorded. Where details of TB diagnoses were missing, these were sought from patient records, clinic TB registers and the Cape Town electronic TB register. Other outcomes recorded were pre-ART death or deferral of ART treatment due to patient ineligibility for ART, treatment refusal or transfer to another ART service or loss to follow-up as described previously [19].

### Statistical analyses

Patient characteristics were summarised using simple descriptive statistics. In all analyses, patients were grouped according to whether the patients had TB diagnoses made in TB clinics and were then referred to the ART clinic, or whether TB was diagnosed in the ART clinic during the pre-ART screening period. Data collected between 2002 and 2008 were aggregated into three sequential calendar periods, 2002-2005, 2005-2006 and 2006-2008. The rationale for this was that 2005 was the year that provider-initiated HIV testing and counselling was instituted in TB clinics and aggregation into three periods provided adequate numbers of patients for the statistical analyses.

**Figure 1:**
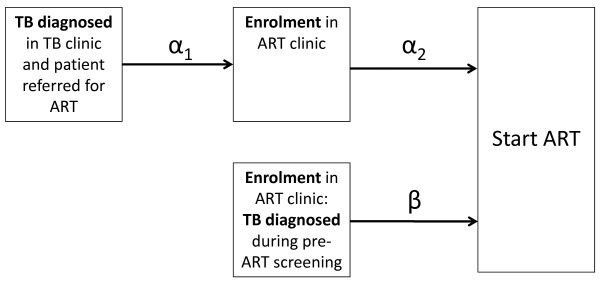
**Conceptual diagram showing the component time periods (α_1_, α_2 _and β) between tuberculosis (TB) diagnosis and starting antiretroviral therapy (ART) for patients with TB diagnosed either in a TB clinic or in the ART clinic**.

The outcomes of primary interest were the time from the start of TB treatment to start of ART and, for patients referred from TB clinics, the two component time periods from TB diagnosis to enrolment in the ART clinic (α_1_) and from enrolment to starting ART (α_2_) (Figure 1). For patients with TB diagnosed in the ART clinic, the delay in ART was a single period referred to as β.

Time delays to starting ART for the subset of 776 patients who did start ART were summarized using medians and interquartile ranges and compared using the Wilcoxon test. Time to starting ART was further analysed using a competing risks regression model [20] that treated pre-ART death and deferral as competing risks to starting ART and included age, gender, calendar period, CD4 near time of TB diagnosis, and TB classification in addition to the group as defined by place of diagnosis. The crude (univariate) and adjusted (multivariate) sub-hazard ratios (SHR) from these models are reported along with their associated 95% confidence intervals (CI) and p-values. The two groups were compared graphically using cumulative incidence functions.

The component periods α_1 _and α_2 _(Figure 1) were also compared using competing risk models, adjusted for the covariates mentioned above. The study population was then stratified according to group, duration of TB treatment prior to ART initiation and CD4 count and compared using a Chi-square test.

## Results

### Patient characteristics and outcomes

Data were available from all 893 consecutive patients with TB who enrolled in the service during the study period and were included in this analysis. Of these, 675 (75.6%) had TB diagnosed in TB clinics and were subsequently referred to the ART clinic. The remaining 218 (24.4%) patients were already enrolled in the ART clinic and had TB diagnosed during the pre-ART screening period. The characteristics of the two groups of patients were similar (Table 1). Patients had advanced immunodeficiency with a median CD4 cell count of 81 cells/μL (IQR, 35-147). TB was sputum smear-positive pulmonary disease in 24% of cases and 30% of all cases were recurrent disease. ART was subsequently started by 776 (87%) patients and, of the remainder, 53 (6%) patients died pre-ART and treatment was deferred in 64 (7%). Comparing patients referred to the ART clinic with TB to those with TB diagnosed in the ART clinic, the proportions who died pre-ART (6% versus 6%) and the proportions who were deferred (5% versus 8%) were similar.

**Table 1:** Characteristics of patients with tuberculosis (TB) stratified according to place of diagnosis

	Patients referred from TB clinic (n = 675)	Patients with TB diagnosed in ART clinic (n = 218)	Total patients (n = 893)
Age, median (IQR)	33 (29-39)	33 (28-39)	33 (29-39)
Female	382 (57%)	142 (65%)	524 (59%)

CD4 cell counts,^b^			
Median (IQR)	81 (37-149)	83 (33-140)	81 (35-147)
0-49	191 (33%)	73 (35%)	264 (34%)
50-99	144 (25%)	46 (22%)	190 (24%)
100-149	103 (17%)	44 (22%)	147 (19%)
150-199	75 (13%)	27 (13%)	102 (13%)
≥ 200	69 (12%)	17 (8%)	86 (11%)

WHO clinical stage			
3	307 (45%)	107 (49%)	414 (46%)
4	368 (55%)	111 (51%)	479 (54%)

Diagnoses made during three calendar periods			
July 2002 - Aug 2005	236 (35%)	70 (32%)	306 (34%)
Sept 2005 - Aug 2006	227 (34%)	73 (34%)	300 (34%)
Sept 2006 - Jan 2008	212 (31%)	75 (34%)	287 (32%)

TB classification ^a^			
Smear-negative pulmonary	224 (33%)	79 (36%)	303 (34%)
Smear-positive pulmonary	162 (24%)	53 (24%)	215 (24%)
Extra-pulmonary	289 (43%)	86 (40%)	375 (42%)
Retreatment TB	185 (27%)	81 (37%)	266 (30%)

^a ^Patients with both pulmonary and extrapulmonary TB were classified as having extrapulmonary disease. Patients with no smear and not having extra-pulmonary TB were classified as smear-negative pulmonary TB

^b ^Data available for 789 (88.4%) patients: 209 (95.0%) of patients diagnosed with TB after enrolment and 582 (86.2%) of patients diagnosed before enrolment.

### Delays in starting ART

**Figure 2:**
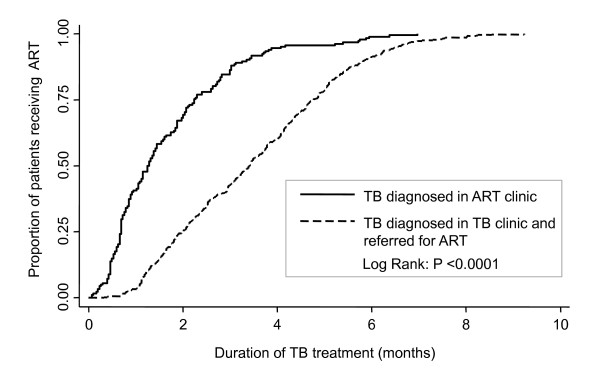
**Time to event analysis showing the cumulative proportion of patients starting antiretroviral therapy (ART) during tuberculosis (TB) treatment**. Data are stratified according to whether the patients were referred from a TB clinic or whether the TB was diagnosed in the ART clinic.

The overall delay between starting TB treatment and starting ART was 95 days (IQR, 49-155). Only 29% of patients started ART within 8 weeks and 44% within 12 weeks (Table 2). Delays were much more prolonged for patients referred from separate TB clinics compared to those in whom TB was diagnosed in the ART clinic (Figure 2) with median delays of 116 days and 41 days, respectively (Table 2). Of patients referred from separate TB clinics, only 19% and 34% started ART within 8 and 12 weeks, respectively. This compares with 59% and 77%, respectively, of those with TB diagnosed within the ART clinic (P < 0.001).

**Table 2:** Time delays between starting TB treatment and starting of antiretroviral therapy (ART) among patients (n = 776) who started treatment.

	Patients referred from TB clinic (n = 581)	Patients with TB diagnosed in ART clinic (n = 195)	All patients (n = 776)
Delay between starting TB treatment and starting ART			
Median (IQR) (days)	116 (68-169)	41 (21-82)	95 (49-155)
< 2 weeks	2 (0%)	16 (8%)	18 (2%)
< 4 weeks	14 (2%)	71 (36%)	85 (11%)
< 6 weeks	62 (11%)	98 (50%)	160 (21%)
< 8 weeks	109 (19%)	115 (59%)	224 (29%)
< 12 weeks	195 (34%)	150 (77%)	345 (44%)
More than 12 weeks	386 (66%)	45 (23%)	431 (56%)

Median (IQR) time from TB diagnosis to ART clinic enrolment (α_1_) (days)	69 (29-115)	-	-

Median (IQR) time from ART clinic enrolment to ART start (α_2_) (days)	32 (28-51)	-	-

We next examined the component delays (α_1 _and α_2; _Figure 1) associated with referral from a TB clinic. The median delay between the start of TB treatment and enrolment in the ART service was more than two-fold longer than the time from enrolment to start of ART (69 days versus 32 days, respectively; P < 0.001) (Table 2). However, once enrolled in the ART clinic, the median time to initiation of ART was similar to that of patients whose TB was diagnosed in the ART service (32 days versus 41 days; P = 0.326).

### Risk factors associated with time to start of ART

We next explored the associations between patient characteristics and the overall time to start of ART from the start of TB treatment (Table 3). In both crude and adjusted analyses, shorter time to ART was strongly associated with treatment in more recent calendar periods, lower CD4 cell counts and smear-positive and extrapulmonary TB. In addition, the time to starting ART was substantially longer for patients referred from TB clinics compared to patients with TB diagnosed in the ART clinic (Table 3). However, there was no association with whether the patient had a new diagnosis of TB or they had recurrent disease.

**Table 3:** Competing risks regression analysis of time from TB diagnosis to start of antiretroviral therapy (ART) for all patients with CD4 cell count measurements (n = 789)

Patient characteristics	**Crude SHR**^**1**^	95% CI	**P value**^**1**^	Adjusted SHR	95% CI	**P value**^**2**^
**Age category**			0.514			
18-24	1.06	0.83-1.36	0.617			
25-39	1.10	0.93-1.31	0.251			
≥40	1					

**Gender**						
Female	1.03	0.89-1.19	0.717			
Male	1					

**Calendar period**			0.022			0.013
Jul 2002 - Aug 2005	1			1		
Sept 2005 - Aug 2006	1.20	1.03-1.41	0.019	1.26	1.05-1.51	0.013
Sept 2006 - Jan 2008	1.21	1.02-1.45	0.033	1.26	1.04-1.53	0.016

**CD4 cell count**			< 0.001			0.001
≥200	1			1		
100-199	1.65	1.29-2.11	< 0.001	1.46	1.13-1.88	0.004
50-99	1.92	1.48-2.49	< 0.001	1.83	1.40-2.38	< 0.001
0-49	1.96	1.50-2.54	< 0.001	1.71	1.30-2.26	< 0.001

**TB classification**						
Retreatment case	1					
New case	1.04	0.9-1.21	0.586			

**Type of TB**			< 0.001			< 0.001
Smear-negative PTB	1			1		
EPTB	1.50	1.26-1.79	< 0.001	1.44	1.17-1.76	0.001
Smear-positive PTB	2.82	2.37-3.37	< 0.001	2.49	2.04-3.04	< 0.001

**Place of TB diagnosis**						
TB clinic	1			1		
ART clinic	1.91	1.54-2.37	< 0.001	1.88	1.51-2.34	< 0.001

^1 ^SHR = sub-hazard ratio. Higher ratios denote more rapid initiation of ART.

^2 ^P values in line with the heading of each variable with more than two categories were calculated using the Wald test.

For patients referred from TB clinics, separate analyses were done to examine factors associated with delays in the referral process (α_1_) and delays within the ART clinic (α_2_). In adjusted analyses, α_1 _delays decreased substantially in more recent calendar periods and were shorter for patients with smear-positive TB but there was no significant association with CD4 cell counts (data not shown). In marked contrast, α_2 _delays were not associated with calendar period but were substantially shorter for patients with lower CD4 cell counts.

### Delays in the most recent calendar period

Since delays in ART decreased in later calendar periods and since those with CD4 cell counts < 50 cells/μL derive the greatest benefit from early ART initiation, we examined delays in the most recent period (2006-2008) stratified by CD4 cell count (Table 4). For patients referred from TB clinics, 36.5% of patients started ART within 8 weeks compared to 63.2% of those with TB diagnosed in the ART clinic (P < 0.001). The corresponding proportions of patients with CD4 cell counts < 50 cells/μL who started ART within one month were 11.1% and 54.6%, respectively (P < 0.001).

**Table 4:** Numbers (proportions) of patients treated in the most recent calendar period (2006-2008) that started antiretroviral therapy (ART) within the first 2, 4, 6 or 8 weeks or after more than 8 weeks of TB treatment.

Duration of TB treatment prior to starting ART	Patients referred from TB clinics		Patients with TB diagnosed in ART clinic	
	
	All patients (n = 171)	Patients with CD4 < 50 (n = 45)	All patients (n = 68)	Patients with CD4 < 50 (n = 22)
	n (%)	n (%)	n (%)	n (%)
< 2 weeks	1 (0.6)	1 (2.2)	6 (8.8)	3 (13.6)
< 4 weeks	6 (3.5)	5 (11.1)	25 (36.8)	12 (54.5)
< 6 weeks	31 (18.1)	20 (44.4)	36 (52.9)	18 (81.8)
< 8 weeks	62 (36.3)	31 (68.9)	43 (63.2)	20 (90.9)
< 12 weeks	98 (57.3)	38 (84.4)	54 (79.4)	21 (95.5)
More than 12 weeks	73 (42.7)	7 (15.6)	14 (20.6)	1 (4.5)

## Discussion

In this study, we found that time period between starting TB treatment and starting ART at this clinic was prolonged and that many patients were not treated within the time-frames recommended under WHO and South African guidelines that were current during the study period. Less than half of the patients started ART within 3 months of TB diagnosis. Most striking was the observation that the median delay was almost three-fold longer for patients who had to be referred for ART from TB clinics located elsewhere in the township compared to patients who had TB diagnosed in the ART clinic. This was not explained by differences in patient characteristics but was instead associated with lack of integration of TB and ART services. In the most recent calendar period, only 11.1% of patients with the highest mortality risk (CD4 cell counts < 50 cells/μL) were referred from TB clinics and received ART within 4 weeks of TB diagnosis compared to 54.6% of similar patients diagnosed in the ART clinic. For referred patients, a majority of the overall delay occurred between TB diagnosis and enrolment in the ART clinic (α_1_). Non-integration of TB and ART services is likely to represent a major obstacle to timely initiation of ART in patients with HIV-associated TB and this may obstruct progress towards reducing mortality in this patient group.

The poor prognosis of patients with HIV-associated TB is transformed by ART and yet mortality in resource-limited settings is nevertheless very high both before and during the initial weeks of ART [21-23]. In light of WHO guidelines revised in 2010 and data from randomised controlled trials, it is now clear that ART must be started much earlier than has likely been widely practised to date. The successful rapid scale-up of ART in resource-limited settings has been achieved through implementation of highly vertical ART programme. However, an unfortunate consequence of this is that in countries such as South Africa, TB treatment and ART have hitherto been largely delivered in separate non-integrated services and this is likely to be a substantial barrier to achieving optimal care including early initiation of ART. This will adversely affect those who have the highest mortality risk who need ART most urgently. This has been recently defined by randomised controlled trials as treatment within 2 weeks for those with CD4 cell counts < 50 cells/μL [9,10]. The delay in starting ART may represent an important indicator of quality of care and that could be used for monitoring and evaluation of programmes caring for patients with HIV-associated TB.

The prolonged delays observed for patients referred from TB clinics are likely to reflect the many steps in the care pathway for such patients. In addition to establishing a TB diagnosis and starting TB treatment, such patients require HIV testing, blood CD4 cell count measurement, referral for ART, enrolment in the ART clinic, reassessment for ART eligibility, preparation for ART and finally starting ART. Each of these steps will be associated with delays and losses that we have not quantified in this study and these may also be compounded by important factors such as stigma, fear and lack of resources of time, energy and transportation money to attend clinics that may not be at the same health facility.

As previously reported in a collaborative analysis of data from multiple South African cohorts, the overall time to starting ART was strongly associated with calendar period and with patient CD4 cell counts [24]. However, in the present study we were able to show that the substantial shortening of delays over sequential calendar periods was specifically due to reductions in the referral delay from the TB clinic. Provider initiated counselling and HIV testing was implemented in TB clinics in this district from 2005 onwards with high uptake and it is likely that high coverage and timely HIV testing at TB diagnosis was a key factor underlying this [15]. Current mass campaigns for HIV testing may also result in a greater proportion of patients knowing their HIV status before presenting with HIV-related illnesses. This may further reduce delays or, ideally, permit earlier initiation of interventions such as isoniazid preventive therapy or ART to prevent TB [25].

The delay within ART clinics to starting treatment was strongly associated with CD4 cell counts, showing an appropriate data responsiveness of health-care workers. In contrast, referral delays from TB clinics were not associated with CD4 cell counts and this is likely to reflect lack of ready availability to CD4 count measurements during the study period. This lack of data responsiveness may also have reflected a lack of awareness within TB clinics of the urgency of starting ART early in patients with low CD4 cell counts and of the need for such referrals to be fast-tracked. This is now being remedied locally by implementation of a fast-tracking system using specially designed referral forms. Should point of care CD4 cell count meters become routinely available within the TB clinics, this may also greatly facilitate more rapid referrals.

Strengths of this study include inclusion of a large number of patients, precise delineation of the time delays in ART initiation and associated risk factors, the analysis of temporal trends and the recording of other patient outcomes (pre-ART death and treatment deferral). Weaknesses include the fact that this was a single site study. Only TB patients who enrolled in the ART clinic were eligible for inclusion and losses between the time of TB diagnosis in TB clinics and enrolment in the ART clinic have not been quantified. Any such losses, however, would further strengthen the argument for the need for integration of TB and ART services. Further operational research is needed to define the component delays along the care pathway between each of the necessary steps (TB diagnosis, HIV testing, CD4 count measurement, referral for ART, enrolment for ART, initiation of ART) and how each of these can be reduced.

## Conclusion

These data show that in this district in South Africa where care by TB and ART services was not integrated and was delivered in different localities, potentially adverse delays in starting ART in patients with HIV-associated TB were largely attributable to the prolonged time between diagnosis in the TB clinic and patient access to the ART clinic. These data support the integration of these two services and we suggest that the time to start ART might be used as an important indicator that should be monitored as measures to improve the care of these patients are implemented.

## Conflicts of interests

The authors declare that they have no competing interests.

## Authors' contributions

SDL and RW designed the study and interpreted the data. RK and CM were responsible for data acquisition and data management. LC and FL did the statistical analyses. SDL wrote the manuscript and all authors commented on drafts and approved the final version.

## Pre-publication history

The pre-publication history for this paper can be accessed here:

http://www.biomedcentral.com/1471-2334/11/258/prepub
